# Estimating the delivery costs of COVID-19 vaccination using the COVID-19 Vaccine Introduction and deployment Costing (CVIC) tool: the Lao People’s Democratic Republic experience

**DOI:** 10.1186/s12916-023-02944-1

**Published:** 2023-07-10

**Authors:** Karene Hoi Ting Yeung, Eunkyoung Kim, Wei Aun Yap, Chansay Pathammavong, Lauren Franzel, Yu Lee Park, Peter Cowley, Ulla Kou Griffiths, Raymond Christiaan W. Hutubessy

**Affiliations:** 1 Department of Immunization, Vaccines and Biologicals, World Health Organization, 20, Avenue Appia, 1211 Geneva 27, Switzerland; 2 World Health Organization, Lao People’s Democratic Republic, 125 Saphanthong Road, Unit5, Ban Saphanthongtai, Sisattanak District, P.O.Box 343, Vientiane Capital, Lao People’s Democratic Republic; 3 Quanticlear Solutions Sdn. Bhd, Kuala Lumpur, Malaysia; 4 Mother and Child Health Center, National Immunization Programme, Ministry of Health, Vientiane Capital, Lao People’s Democratic Republic; 5 Department of Health Governance and Financing, World Health Organization, 20, Avenue Appia, 1211 Geneva 27, Switzerland; 6 United Nations Children’s Fund, 3 UN Plaza, New York, NY 10017 USA

**Keywords:** COVID-19, Coronavirus, Vaccine, Costs, CVIC, Lao People’s Democratic Republic

## Abstract

**Background:**

The COVID-19 Vaccine Introduction and deployment Costing (CVIC) tool was developed to assist countries to estimate incremental financial costs to roll out COVID-19 vaccines. This article describes the purposes, assumptions and methods used in the CVIC tool and presents the estimated financial costs of delivering COVID-19 vaccines in the Lao People’s Democratic Republic (Lao PDR).

**Methods:**

From March to September 2021, a multidisciplinary team in Lao PDR was involved in the costing exercise of the National Deployment and Vaccination Plan for COVID-19 vaccines to develop potential scenarios and gather inputs using the CVIC tool. Financial costs of introducing COVID-19 vaccines for 3 years from 2021 to 2023 were projected from the government perspective. All costs were collected in 2021 Lao Kip and presented in United States dollar.

**Results:**

From 2021 to 2023, the financial cost required to vaccinate all adults in Lao PDR with primary series of COVID-19 vaccines (1 dose for Ad26.COV2.S (recombinant) vaccine and 2 doses for the other vaccine products) is estimated to be US$6.44 million (excluding vaccine costs) and additionally US$1.44 million and US$1.62 million to include teenagers and children, respectively. These translate to financial costs of US$0.79–0.81 per dose, which decrease to US$0.6 when two boosters are introduced to the population. Capital and operational cold-chain costs contributed 15–34% and 15–24% of the total costs in all scenarios, respectively. 17–26% went to data management, monitoring and evaluation, and oversight, and 13–22% to vaccine delivery.

**Conclusions:**

With the CVIC tool, costs of five scenarios were estimated with different target population and booster dose use. These facilitated Lao PDR to refine their strategic planning for COVID-19 vaccine rollout and to decide on the level of external resources needed to mobilize and support outreach services. The results may further inform inputs in cost-effectiveness or cost–benefit analyses and potentially be applied and adjusted in similar low- and middle-income settings.

**Supplementary Information:**

The online version contains supplementary material available at 10.1186/s12916-023-02944-1.

## Background

Coronavirus disease 2019 (COVID-19) emerged as a pandemic with major health and economic impacts since December 2019. As of 9 December 2022, there have been 644 million confirmed cases and 6.6 million deaths due to COVID-19 reported to the World Health Organization (WHO), with the reported number of cases ranging from 9.4 million in Africa to 267 million in Europe, and reported number of deaths ranging from 175,000 in Africa to 2.9 million in the Americas [1]. In addition to the public health and social measures, such as frequent hand hygiene, proper use of masks and physical and social distancing, to control the spread of COVID-19 [2], vaccination is an effective way to prevent people from getting severe illnesses or dying from COVID-19 [3]. As of 10 December 2022, 13 COVID-19 vaccine products have obtained the WHO Emergency Use Listing Procedure (EUL) [4].

The rollout of COVID-19 vaccines is different from that of childhood vaccines. The target population is wider since it mainly covers the adult working population and the elderly. The cold-chain system might need to be expanded and more healthcare workers are needed to deliver a higher number of vaccines for these populations, and thus the vaccine delivery costs will be higher. As COVID-19 vaccines are new, vaccine hesitancy will require more efforts for demand generation [5, 6] and thus will incur costs. To facilitate timely access to COVID-19 vaccines, countries should develop comprehensive and feasible National Deployment and Vaccination Plans (NDVP) [7]. Countries receiving vaccine supplies from COVAX, such as the Lao People’s Democratic Republic (Lao PDR), were required to submit their NDVP before vaccines would be granted [8]. Credible estimation of multi-year costs of the NDVP assists resource mobilization and is one of the critical components to ensure its feasibility without compromising existing essential health services.

Total delivery costs for COVID-19 vaccination covering 70% of the population and protecting human resources for essential health services fully in 133 low- and middle-income countries, has been estimated at US$8.4 billion by the COVAX Readiness and Delivery Working Group on Delivery Costing [9]. There are still only few published studies on COVID-19 vaccine delivery costs or costing processes. There has been a modelling study on cost of vaccine delivery strategies in low- and middle-income countries during the COVID-19 pandemic [10], and cost analyses of COVID-19 vaccine in Ghana [11] and Kenya [12]. Cost-effectiveness studies of COVID-19 vaccines in Denmark [13], Hong Kong [14], Kenya [15], Pakistan [16] and low- and middle-income countries [17] are also available, but there are little details on vaccine delivery costs. Realistic delivery cost estimation can identify the resource needs and gaps to inform domestic resource mobilization and requests for external funding when necessary.

Several tools and templates with different purposes were developed by different organizations during the COVID-19 pandemic. WHO COVID-19 Essential Supplies Forecasting Tool (COVID-ESFT) was developed to assist countries to forecast the necessary resources needed to manage and treat COVID-19, but vaccination was not included [18]. The COVID-19 Vaccine Introduction Readiness Assessment Tool (VIRAT/VRAF 2.0) was a tool to support countries in assessing programme readiness to introduce COVID-19 vaccines and identify financial gaps to optimize vaccine delivery [19]. Gavi and UNICEF have also provided budgeting templates for countries to apply for COVID-19 Delivery Support Fund, which assisted countries in identifying financial gaps for external resources. WHO and UNICEF developed the COVID-19 Vaccine Introduction and deployment Costing (CVIC) tool to help countries estimate the costs to introduce COVID-19 vaccines for budgeting use. The development of the CVIC tool began in the WHO regional office for the Western Pacific (WPRO) and was piloted and validated in WPRO member states, before being adopted at the global level and refined further through the inter-agency working group and other WHO regional offices (see Additional file 1: Appendix 1). The tool is in alignment with the NDVP guidance [7], and operational guides, such as the VIRAT/VRAF 2.0 [19], WHO Strategic Advisory Group of Experts on Immunization (SAGE) roadmap for prioritizing vaccine use [20], and the standard terminology and principles for vaccine delivery costs [21, 22]. The estimates from the tool are compliant with the medium-term expenditure framework format used by Ministries of Finance, which is an approach integrating fiscal objectives and budgeting over a multi-year period [23]. The tool can be used by government programme managers and policy makers to conduct costing exercises and update their vaccination plans in a harmonized way and to obtain a structured and comprehensive estimation of incremental costs required to roll out COVID-19 vaccines. As of 3 November 2022, 20,320 persons from 177 countries have joined the online course of the CVIC tool [22]. At least 25 countries have completed the CVIC tool costing exercise [11]. This article describes the purposes, assumptions and methods used in the CVIC tool and presents the estimated incremental financial costs of introducing COVID-19 vaccines in Lao PDR.

Lao PDR is a lower-middle-income country in Southeast Asia with a 2021 population of 7.3 million people living in Vientiane and 17 provinces [24]. Between 3 January 2020 and 9 December 2022, Lao PDR reported 217,026 confirmed COVID-19 cases and 757 deaths [1]. With experience in getting high coverage for routine vaccination in children and a national-wide vaccination campaign against pandemic influenza virus, Lao PDR was positioned to be prepared to identify target groups for COVID-19 vaccination. As of 20 November 2022, a total of 12.8 million COVID-19 vaccine doses have been administered and 6.1 million persons have been vaccinated with at least one dose [1]. In early 2021, the Ministry of Health (MoH) of Lao PDR initiated the costing exercise for COVID-19 vaccination using the CVIC tool to estimate the costs of the NDVP and to develop a budget for COVID-19 vaccine implementation, resource mapping and strategy setting. Apart from the MoH, the WHO country office took the lead in the costing process in close collaboration with development partners, including the World Bank and UNICEF.

## Methods

### Purposes and features of the CVIC tool

The CVIC tool [25, 26] is a prepopulated, updateable, multi-lingual Microsoft Excel-based tool to estimate and project incremental financial costs needed to introduce COVID-19 vaccines in a country over a 3-year period. It provides cost estimates by budgetary year and could be used for resource mobilization due to its operational nature. The budget implications of capital costs only happen in the year of purchase so the CVIC tool provides non-annualized costs only. Costs are generated from the payers’ perspective, i.e. monetary costs needed to be financed by the government, partners (including non-governmental organizations), and the private sector. The CVIC tool is primarily a costing tool, but it can also help countries refine their delivery strategies for COVID-19 vaccine rollout dynamically by varying inputs and assumptions entered and observing the cost implications of strategy choices. In addition, the tool provides a health systems perspective by summarizing the human resource implications of mass vaccine deployment.

The CVIC tool can project total costs over a 3-year period, by 6-month periods, by cost categories, and by delivery modalities. This facilitates identification of the cost structure of COVID-19 vaccine delivery, including estimation of surge human resource needs. Resource needs can be mapped to potential financing sources. The COVID-19 vaccine database built into the tool includes current vaccine specifications and pricing information of the primary series, which is updatable without downloading a new version of the tool. Thus, users can select new vaccines under the EUL after refreshing the database.

### Steps for conducting COVID-19 vaccine delivery cost projection using the CVIC tool [9]

Ideally, a country’s NDVP should form the basis for the costing exercise. The costing process should involve an in-country multidisciplinary team at national and sub-national level, including professionals from the immunization programme, MoH, Ministry of Finance and persons who are familiar with costing and/or health systems. The team should obtain an initial rollout plan and country-specific data required to complete the CVIC tool. Some of the information should be available in the NDVP. The data required include:

- Target population definition, size and prioritization. High-risk groups are usually targeted and vaccinated first. A SAGE roadmap is available to assist countries in prioritizing the uses of COVID-19 vaccines [20].
- Delivery strategies, including delivery modalities and choice of vaccine types for specific target populations.
- Vaccine supply arrangements, including vaccine types, quantities, prices, and time availability.
- Country-specific unit costs, such as domestic transport and human resource costs.
- Country-specific costs for central-level activities, such as technical assistance and operational expenses.

Country-specific data should be prioritized even though some parameters are prepopulated based on global databases and models (see Additional file 1: Appendix 2). With all inputs being gathered and entered into the CVIC tool, the incremental resource requirements for COVID-19 vaccine introduction will be calculated.

### Delivery modalities in the CVIC tool

Four delivery modalities have been defined in the CVIC tool. The application of a delivery modality depends on the nature of target populations and each delivery modality has implications on delivery costs. Table 1 presents the suggested delivery modalities for different target populations and the hypothesized cost implications of each delivery modality.

**Table 1:** Delivery modalities for COVID-19 vaccination in the CVIC tool [9]

Delivery modality	Examples	Linked target populations	Cost implication
1. Fixed sites with cold storage equipment	Hospitals and health facilities	▪ Healthcare workers ▪ Essential workers and related groups ▪ Older adults (optional to include their household members) ▪ Immunocompromised persons ▪ Groups with comorbidities or health states that put them at increased risk of severe diseases (optional to include their household members) ▪ Disadvantaged socioeconomic groups at increased risk of severe disease or death ▪ Remaining groups	The least costly delivery modality due to: ▪ ability to store vaccines; ▪ no per diems and transport for healthcare workers; and ▪ fewer requirements for demand-generation activities
2. Fixed sites without cold storage equipment	Small health centres, community halls and mass vaccination sites	▪ Essential workers and related groups ▪ Older adults (optional to include their household members) ▪ Immunocompromised persons ▪ Groups with comorbidities or health states that put them at increased risk of severe diseases (optional to include their household members) ▪ Disadvantaged socioeconomic groups at increased risk of severe disease or death ▪ Remaining groups	More costly than modalities 1 and 3 due to: ▪ transport of vaccines in cold boxes and vaccine carriers; ▪ per diems and transport required for healthcare workers; and ▪ some demand-generation activities required for information of venues and times for vaccination
3. Residential institutions	Elderly homes and refugee camps	▪ Older adults and staff in residential institutions ▪ Disadvantaged socioeconomic groups at increased risk of severe disease or death and staff in residential institutions	More costly than modality 1 due to: ▪ transport of vaccines in cold boxes and vaccine carriers; and ▪ per diems and transport required for healthcare workers Less costly than modality 2 due to: ▪ fewer demand-generation activities for less mobile populations
4. Outreach with overnight stays for healthcare workers and mobile vaccination	Outreach to hard-to-reach groups	Hard-to-reach populations	The most costly delivery modality due to: ▪ transport of vaccines in cold boxes and vaccine carriers; ▪ most per diems and transport required for healthcare workers for overnight stays; and ▪ most intensive demand-generation activities required for communicating benefits of vaccination

### Cost components in the CVIC tool

The cost components and activities in the CVIC tool are aligned with the NDVP guidance for COVID-19 vaccines [7]. It covers nine main categories that includes recurrent (operational) costs and capital costs needed for COVID-19 vaccine deployment (Table 2).

**Table 2:** Cost categories for COVID-19 vaccination included in the CVIC tool

Cost category	Descriptions
1. Cross-cutting technical assistance for planning, coordination and delivery	Technical assistance for planning, coordination, costing, budgeting, financing, regulatory preparedness, identification of target population and prioritization, disease surveillance, service delivery, microplanning, vaccine procurement, cold chain requirements, planning for logistics and infrastructure
2. Vaccine doses and related devices and supplies	Vaccine doses
	Vaccine-related supplies, such as syringes and safety boxes
3. Vaccinators (healthcare workers)	Technical assistance
	Healthcare worker training and supervision
	Healthcare worker compensation, including supplementary wages, allowances for mobile teams and salaries for additional human resources for health
4. Vaccine delivery	Logistics and transportation related to delivery strategies, including outreach and mobile teams such as fuel for distributing vaccines, airfreight, handling costs, clearance and procurement fees
	Waste management
	Personal protective equipment
	Security costs for vaccine transportation and during vaccine administration
5. Cold chain	Freezers, refrigerators and cold boxes
	Operational expenses to distribute cold-chain equipment, storage and transportation, including staff, infrastructure, energy, tracking and monitoring stock through the vaccine logistics management and information system
6. Data management, monitoring and evaluation and oversight (for both electronic and paper-based data management and monitoring systems)	Technical assistance
	Operational expenditures
	Evaluation including studies related to vaccine introduction, costing, coverage and effectiveness
	Oversight and quality assurance
7. Vaccine safety surveillance and injection safety (including reporting of adverse events following immunization, investigation, causality assessment and responses)	Technical assistance
	Operational expenditures such as for home-based records, registers of vaccinated persons and tally sheets, vaccine logistics management information systems and health information
	Systems used to gather, monitor, evaluate, analyse, produce and disseminate information across traditional and non-traditional providers
	Compensation schemes for adverse events following immunization
8. Demand generation and communications (risk communication and community engagement)	Technical assistance
	Operational expenditures to support vaccine uptake and acceptance, including social listening, data collection and analysis, use of local behavioural and social data, social mobilization, crisis communications, operating social listening systems, rumour management, assessing behavioural data, risk communication and community engagement, mass media, and printing posters and banners
9. Protecting essential health services and health systems strengthening	Co-delivered activities and interventions that are not specific to COVID-19 vaccination, but are intended to strengthen health systems or protect essential health services, or both

### A case study for Lao PDR

#### Data sources

From March to September 2021, the costing team in Lao PDR collected data from different information sources (Table 3). The results presented in this article were from the CVIC tool version 2.3. Lao PDR projected incremental financial costs needed to introduce COVID-19 vaccines for 3 years from 2021 to 2023 from the government perspective. Since almost all COVID-19 vaccines during the study period (2021–2023) were projected to be donated and some COVAX vaccines were purchased using the World Bank loan, the costs reported in this article exclude vaccine costs. Both non-annualized and annualized (over ten expected useful life years) costs were presented in this article. All costs were collected in 2021 Lao Kip and presented in United States dollar (US$) using an exchange rate of 11,718.75 Kip to one US$ [27].

**Table 3:** Key inputs used and data sources for COVID-19 vaccine introduction delivery cost estimates in Lao PDR

Data	Assumption	Source
Total population	2021: 7,337,714 2022: 7,442,794 2023: 7,545,792	Bureau of Statistics, Lao PDR
Number of distribution points	Provincial/central levels: 26 District levels: 135	EPI, Lao PDR
Number of service points	Fixed sites with cold storage equipment: 198 Campaign sites: 1645 for adults and 8813 for children Outreach sites: 5081	EPI, Lao PDR
Vaccine products	Ad26.COV2.S (recombinant), VAXZEVRIA, COMIRNATY, Vero Cell	EPI, Lao PDR
Expected willingness to receive COVID-19 vaccines	100%	Assumption with agreement with EPI, Lao PDR
Vaccine wastage rate	5%	Assumption with agreement with EPI, Lao PDR
Maximum number of redeployable human resources for health	6807 (including medical students and private sector)	EPI, Lao PDR
Robustness adjustment for human resources for health^a^	2.5 min	CVIC default
Per diem for healthcare workers for outreach (daily rate)	US$2.56	Document on MoH regulation
Cost of vaccinator training and supervision	US$80,055	Programme managers of MoH, Lao PDR
Cost of personal protective equipment per healthcare worker per day	US$3.66	Programme managers of MoH, Lao PDR
Transportation cost for healthcare workers	US$2.56 per motorcycle per day	Document on MoH regulation, Lao PDR
Cost per litre for additional ultra-cold storage	US$8.6	Programme managers of MoH, Lao PDR
Costs per trip for cold-chain logistics and domestic transport from central to regional	Refrigerated: US$100 Ultra-cold: US$100	CVIC default based on global vaccine costing derived from global empirical studies and confirmed by experts in Lao PDR
Costs per trip for cold-chain logistics and domestic transport from regional to districts and fixed sites	Refrigerated: US$50 Ultra-cold: US$50	CVIC default based on global vaccine costing derived from global empirical studies and confirmed by experts in Lao PDR
Cost per fixed site or team for IT infrastructure per fixed site or team for local data management and monitoring	US$1250	Expert opinions from the consultation in Lao PDR
Cost per site per day for stationery and data charges for telecommunications per site per day	US$3.41	Expert opinions from the consultation in Lao PDR
Cost per site for materials and media costs per site per local demand generation and communications	US$38.29	Expert opinions from the consultation in Lao PDR

All costs are in 2021 United States dollars (US$)

*CVIC* COVID-19 Vaccine Introduction and deployment Costing Tool, *EPI* Expanded Programme on Immunization, *Lao PDR* the Lao People’s Democratic Republic, *MoH* Ministry of Health

^a^Minutes of healthcare worker time required to vaccinate one dose to be added to (upper variant) or subtracted from (lower variant) the medium variant

#### Costing scenarios, vaccine delivery strategies and other assumptions

Lao PDR targeted to vaccinate 80% of its entire population with the primary series by 2022, including priority target population (i.e. healthcare workers, older adults aged above 60 years, persons with underlying conditions and essential workers), adults, teenagers and children aged 5 years and above; and to vaccinate 50% of its population with an initial booster dose by 2023. All adults and teenagers are also offered to receive second booster doses. In the cost analysis, 5 scenarios with different coverage goals for the target population groups were generated (Table 4).

**Table 4:** Costing scenarios of COVID-19 vaccine introduction in Lao PDR

Scenario		Percentage of total population vaccinated with a primary series or booster of COVID-19 vaccine (%)							
			**2021–2023**	**H1 2021**	**H2 2021**	**H1 2022**	**H2 2022**	**H1 2023**	**H2 2023**
S1	Primary series for the priority population^a^ and all adults	Total	63	8	42	10	3	0	0
S2	Primary series for the priority population^a^, all adults and** teenagers**	Additional to S1	14	0	2	5	5	2	0
		Total	77	8	44	15	8	2	0
S3	Primary series for the priority population^a^, all adults, teenagers and **children aged 5–11 years**	Additional to S2	17	0	0	8	7	2	0
		Total	94	8	44	23	15	4	0
S4a	Primary series and **one initial booster** for the priority population^a^, all adults and teenagers	Additional to S2	77	0	8	44	15	8	2
		Total	154	8	52	59	23	10	2
S4b	Primary series and **two booster doses** for the priority population^a^, all adults and teenagers	Additional to S4a	75	0	0	0	8	44	23
		Total	229	8	52	59	31	54	25
S5a	Primary series and **one initial booster** for the priority population^a^, all adults, teenagers and **children**	Initial booster for children	17	0	0	0	8	7	2
		Total	188	8	52	67	38	19	4
S5b	Primary series and **two booster doses** for the priority population^a^, all adults, teenagers and **children**	Second boost for children	15	0	0	0	0	8	7
		Total	278	8	52	67	46	71	34

H1, first half of the year; H2, second half of the year

^a^Priority population includes healthcare workers, older adults aged above 60 years, persons with underlying conditions and essential workers

Vaccination is conducted through a combination of fixed sites with cold storage equipment, campaign sites without cold storage equipment and community outreach sites. Fixed vaccination sites are established at the central, provincial and district levels, health centres and hospitals. Temporary sites are established for mass vaccination campaign. Outreach sessions are delivered through district hospitals and health centres.

Four available vaccines were included: Ad26.COV2-S (recombinant), COMIRNATY, VAXZEVRIA and Vero Cell. Since only limited quantities of CoronaVac and Sputnik V have been available in Lao PDR, these were excluded from the analysis.

## Results

**Fig. 1:**
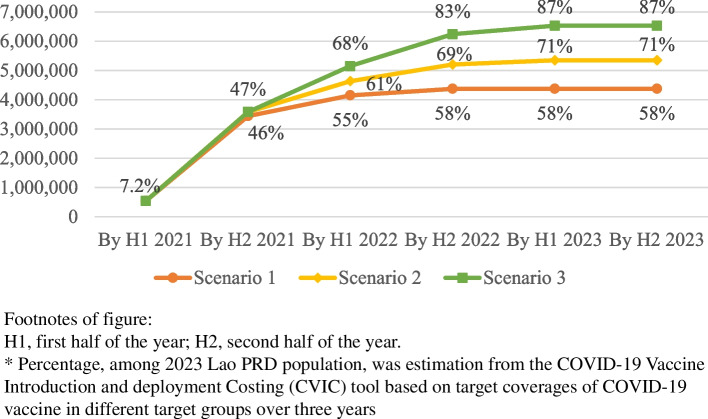
Cumulative person counts and percentages* of completed primary courses of COVID-19 vaccine in Lao PRD

With 100% of the target population expected to be willing to receive COVID-19 vaccines, 7.2% of 2023 Lao PDR population was expected to be vaccinated with the primary courses of COVID-19 vaccines in the first half of 2021 (Fig. 1). By 2023, 58, 71 and 87% of population were projected to receive the primary courses in scenarios 1, 2 and 3, respectively.

**Fig. 2:**
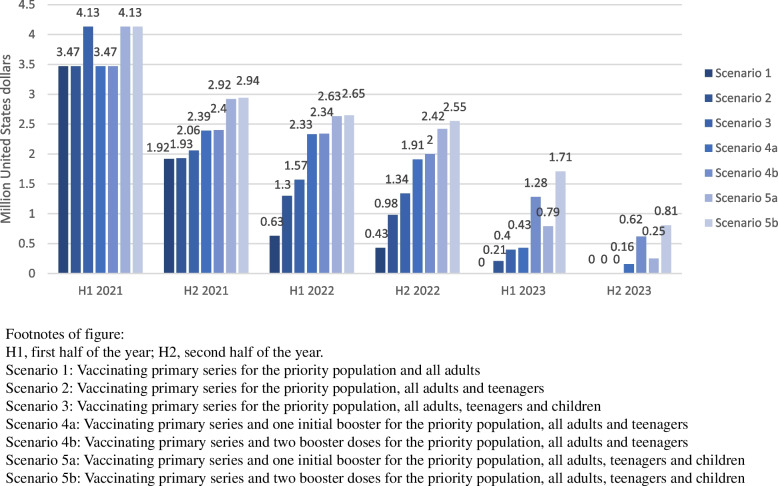
Total non-annualized costs of COVID-19 vaccine introduction (excluding vaccine costs) in Lao PDR by scenarios over 2021–2023

It was estimated that the government of Lao PDR needs a total non-annualized financial cost of US$6.44 million to vaccinate all adults (scenario 1) with COVID-19 vaccine in 2021–2023 (excluding vaccine costs), additionally US$1.44 million and US$1.62 million for teenagers (scenario 2) and children (scenario 3), respectively (Table 5). Providing one and two boosters to all adults and teenagers are estimated to increase the total non-annualized financial cost from US$7.88 million (scenario 2) to US$10.69 million (scenario 4a) and US$12.11 million (scenario 4b), respectively. However, the cost per dose will be lowered from US$0.81 (scenario 2) to US$0.71 (scenario 4a) and US$0.6 (scenario 4b) respectively. Vaccinating all children with one and two boosters are estimated to increase the total non-annualized financial cost from US$9.5 million (scenario 3) to US$13.14 million (scenario 5a) and US$14.8 million (scenario 5b) respectively and lower the cost per dose from US$0.79 (scenario 3) to US$0.71 (scenario 5a) and US$0.6 (scenario 5b) respectively. In all scenarios, the non-annualized costs are projected to be the highest in the first half of 2021 (initial phase of vaccine introduction) and gradually decrease over time (Fig. 2). When the capital costs of cold-chain expansion were annualized over the expected useful life of 10 years, the costs peaked in the second half of 2021.

**Table 5:** Costing summary of COVID-19 vaccine introduction in Lao PDR in 2021–2023 (2021 United States dollars)

	Scenario 1	Scenario 2	Scenario 3	Scenario 4a	Scenario 4b	Scenario 5a	Scenario 5b
**Total number of vaccine doses administered**	7,997,333	9,736,171	12,036,265	15,091,435	20,316,692	18,560,415	24,832,693
**Total number of vaccine courses administered**	4,374,387	5,350,000	6,527,972	10,705,264	15,930,521	13,052,122	19,324,400
**Total annualized cost (million dollars)**^ab^	4.91	6.35	7.96	9.16	10.57	11.6	13.25
**Total non-annualized cost (million dollars)**^a^	6.44	7.88	9.5	10.69	12.11	13.14	14.8
**Cost per dose (non-annualized)**^a^	0.81	0.81	0.79	0.71	0.6	0.71	0.6
**Cost per primary course (non-annualized)**^**a**^	1.47	1.47	1.46	1	0.76	1.01	0.77

Scenario 1: Vaccinating primary series for the priority population and all adults

Scenario 2: Vaccinating primary series for the priority population, all adults and teenagers

Scenario 3: Vaccinating primary series for the priority population, all adults, teenagers and children

Scenario 4a: Vaccinating primary series and one initial booster for the priority population, all adults and teenagers

Scenario 4b: Vaccinating primary series and two booster doses for the priority population, all adults and teenagers

Scenario 5a: Vaccinating primary series and one initial booster for the priority population, all adults, teenagers and children

Scenario 5b: Vaccinating primary series and two booster doses for the priority population, all adults, teenagers and children

^a^Costs presented exclude vaccine costs

^b^Annualized over 10 useful life years

**Fig. 3:**
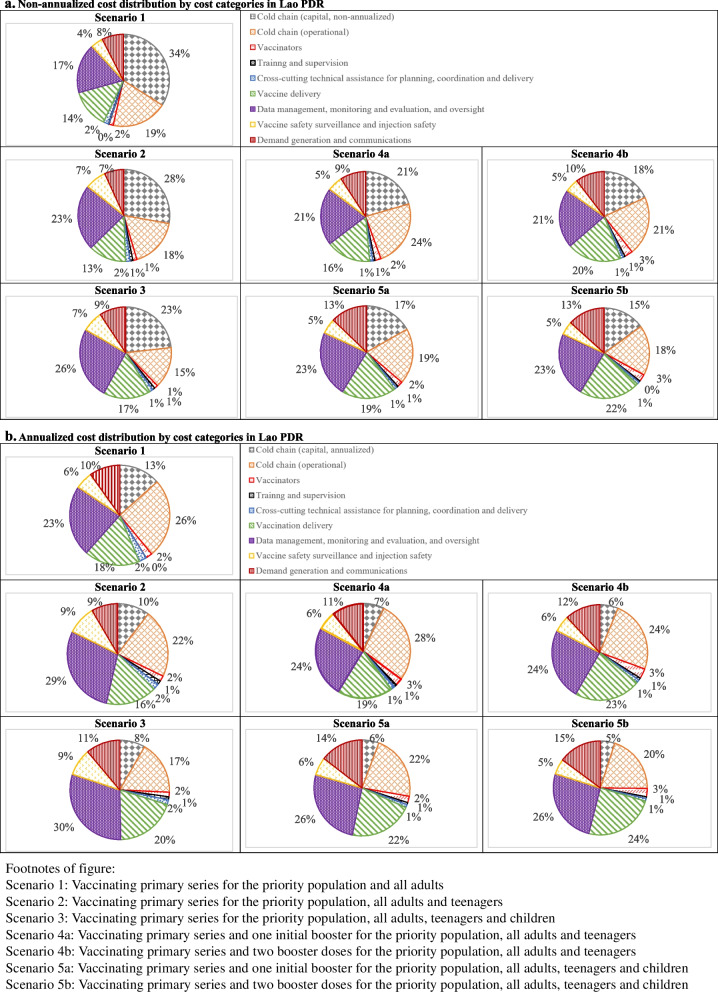
**a** Non-annualized cost distribution by cost categories in Lao PDR. **b** Annualized cost distribution by cost categories in Lao PDR

When capital costs are not annualized, the capital expenditure of cold chain contributed most to vaccinate adults (scenario 1, 34%) and teenagers (scenario 2, 28%) with primary series of COVID-19 vaccines (Fig. 3a). Costs on data management, oversight, monitoring and evaluation become the cost driver when vaccinating children (scenario 3, 25%). When the capital costs are annualized, the operational expenditure of cold chain (scenario 1, 26%) and costs on data management, oversight, monitoring and evaluation (scenarios 2 and 3, 29–30%) drove the costs (Fig. 3b). If booster doses are introduced, regardless of the annualization, data management, oversight, monitoring and evaluation contributed most when children are vaccinated (23–26%) and operational expenditure of cold chain contributed most when children are not covered (21–24%). There is no expected spending on protecting essential health services and health systems strengthening. Apart from this, costs on human resources (per diems to healthcare workers) (1–3%) and training and supervision (0–1%) contributed least since all healthcare workers are redeployed and no new human resources is needed to be hired, and per diem rates were conservatively applied.

It is estimated that the number of new cold storage equipment required for scenarios 1–3 is the same: one walk-in cold room, 610 freezers/refrigerators and 438 remote temperature monitoring devices. If boosters are introduced, additional equipment would be needed: one walk-in cold room, 558–762 freezers/refrigerators and 386–590 remote temperature monitoring devices.

**Fig. 4:**
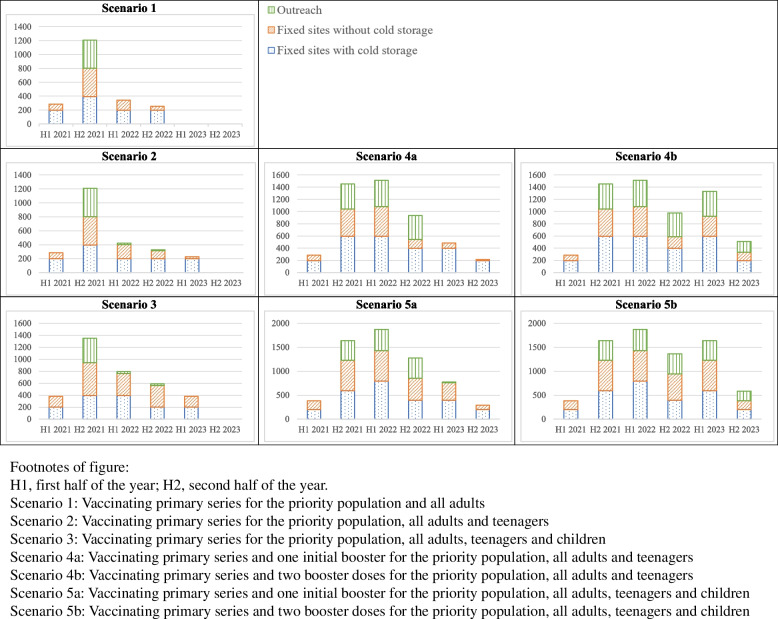
Number of healthcare workers required by delivery modality in Lao PDR

The total number of healthcare workers required in the first 3 years of COVID-19 vaccine introduction in Lao PDR ranged from 2094 in scenario 1 to 7477 in scenario 5. The highest number of healthcare workers required was 1871 in the first half of 2022 in scenario 5. Figure 4 shows the number of healthcare workers required by the three delivery modalities over time in different scenarios.

## Discussion

Using the CVIC tool, Lao PDR has costed five scenarios of COVID-19 vaccine introduction with non-annualized, incremental, financial costs ranging from US$6.44 million to US$14.80 million, equivalent to 1.32 to 3.04% of the total government health expenditure of Lao PDR in 2019, and 0.03 to 0.08% of Lao PDR’s gross domestic product [28]. Including teenagers and children in the vaccination programme increases the coverage from 58 to 70.9% and 86.5%, respectively, and introducing two booster doses decreases the cost per dose from US$0.8 to US$0.6.

Since no new healthcare workers were needed to be hired, based on NDVP, and the per diem rates were conservatively applied, the financial cost on human resources from this cost analysis were relatively small. However, it is also worth noting that there are opportunity costs in redeploying existing healthcare workers. As expected, increasing the vaccination coverage, the costs of vaccine delivery and demand generation and communications increased. Comparing to vaccinating adults only, introducing COVID-19 vaccine to teenagers would increase the costs on oversight and assurance, and vaccine safety and surveillance. More oversight and more resources on vaccine safety surveillance are needed to vaccinate teenagers.

Capital spending on cold storage drove the costs if it was not annualized. However, the refrigerators can be used for more than a year. When these costs were annualized, operational expenditure on cold chain and costs on data management, oversight, monitoring and evaluation played a larger contribution to the total costs. Also, the refrigerators can also be used to store other vaccines but shared costs were not considered so the cost estimated may be overestimated.

The majority of the primary courses were administered in the second half of 2021 so manpower required for primary courses also peaks at the time. If booster doses are introduced, the number of healthcare workers required would peak in the first half of 2022. Most healthcare workers were allocated to work at fixed sites with cold storage equipment for adults and teenagers. After introducing the vaccine to children, more healthcare workers would be needed for fixed sites without cold storage equipment. When boosters were introduced, the needs of outreach increased.

The estimated COVID-19 vaccine delivery costs per primary course in Lao PDR (US$0.6–0.81) were lower than those in Ghana (US$4.5–4.6) and Kenya (US$4.28–3.29), where published comprehensive costing was available [11, 12]. The per diem for one healthcare worker, unit costs of personal protective equipment and transportation in Ghana were higher than those in Lao PDR but Lao PDR has higher unit costs of local data management and monitoring, and demand generation and communication. The numbers of healthcare workers required per primary course were similar in Ghana and Lao PDR.

A comprehensive costing exercise of a new vaccine introduction can be useful, especially during public health emergencies. Vaccine procurement cost usually drives the vaccination programme cost, but obtaining vaccine delivery cost estimates can help refine delivery strategies, such as coverage goal, target population, delivery modalities, and minimize the financial needs and burden to the existing health systems, with a better budgeting plan at an initial phase of the deployment process. The CVIC tool provides a list of data required and prepopulated data as reference for countries to obtain cost estimates in a structured way for their effective and timely planning and resource mobilization, and allows for the incorporation of different delivery scenarios by cost driver typology, whether it be by capital, human resource or other recurrent variables, such as training, service delivery, monitoring, etc. Countries can use the tool to fit their needs such as seen in the case in Lao PDR where there was a request to estimate the costs of boosters; for scenario analyses to determine an appropriate vaccination strategy; and for costing out the NDVP. In Lao PDR, the cost estimates from the CVIC tool were used as inputs for the NDVP, and to inform national level planning and resource allocation. Lao PDR eventually decided to vaccinate primary series and one initial booster for the priority population (including healthcare workers, older adults aged above 60 years, persons with underlying conditions and essential workers), all adults, teenagers and children aged 5 years and above; and second booster doses for all adults and teenagers aged 12 years and above. The tool also helped the government understand the COVAX policy environment including potential cost sharing scenarios. Based on the cost scenarios developed in the tool, the government took decisions on the level of external resources that needed to mobilize and to support outreach services.

The CVIC tool can aid in budgeting but also requires contribution from local authorities or experts on formulating efficient strategies in a local setting. The delivery strategies for COVID-19 vaccination vary among countries and different delivery modalities would have different cost impacts and thus different implication to the immunization programme and health system. Lao PDR vaccinates their target groups through fixed vaccination sites with and without cold storage equipment and outreach sites. Some countries in Latin America deliver COVID-19 vaccines to the high-risk groups at the same time as influenza vaccine through the already established programmes [29]. In this situation, some costs could be shared with the influenza vaccination programme. Other countries, particularly in Africa, rely on campaigns to deliver COVID-19 vaccines [30], which would increase the costs of per diems and transport for healthcare workers. The Project Last Mile, a partnership between Coca-Cola company, donors and the governments in Africa, is an example of reducing costs and increasing efficiency of vaccine delivery in lower- and middle-income settings. It helped accelerate COVID-19 vaccine distribution and communications in Africa vaccines using their logistics network and supply chain [31].

The CVIC tool version 2.3 has several limitations. First, it is not a micro-costing tool—sub-national specificity is not captured even if there is sub-national variation, for example, in the population size of provinces or districts. Second, booster doses cannot be easily captured in the tool. A separated Excel workbook of the tool can be an option for the booster dose. Third, although a combination of vaccine products can be selected for one delivery modality, similar characteristics of vaccine products are required such as the number of doses and cold storage requirement. In a costing exercise of COVID-19 vaccines in general, the difficulty in obtaining all necessary data limits the accuracy of the projection. In addition, conducting the costing exercise at the same time with planning and rolling out the vaccines under a pandemic is also very challenging. There are many uncertainties that may lead to a change of the vaccination plan. Model input uncertainty should be handled with precaution. A sensitivity analysis on varying lower and upper values of parameters would help in understanding the cost drivers. The CVIC tool can produce upper and lower uncertainty bounds for the cost estimates by varying the healthcare worker time required to vaccinate one dose. Also, the costing results may be difficult to catch up with the reality. This also applies to the case in Lao PDR. For the cost analysis in Lao PDR, the expected willingness to receive the vaccines is 100% which may be difficult to achieve in reality. Last but not least, a cost analysis cannot provide insight on the health impact of the vaccine. A cost-effectiveness or cost–benefit analysis would be useful to explore the incremental cost to gain a unit of a health outcome to support decision-making.

## Conclusions

With the CVIC tool, this study estimated costs of five scenarios with different target population and booster use in Lao PDR. These facilitated Lao PDR to refine their strategic planning for COVID-19 vaccine rollout and to decide on the level of external resources needed to mobilize and to support outreach services. The results may further inform inputs in cost-effectiveness or cost–benefit analyses and potentially be applied and adjusted in similar low- and middle-income settings.

## Supplementary Information

**Additional file 1: Appendix 1.** Timeline for the development and key milestones of the COVID-19 Vaccine Introduction and deployment Costing (CVIC) tool. **Appendix 2.** Prepopulated data and sources in the COVID-19 Vaccine Introduction and deployment Costing (CVIC) tool.

## Data Availability

The data are accessible to researchers upon reasonable request for data sharing to the corresponding author. Requests for data require approval by the MoH of Lao.

## Abbreviations

COVID-19: Coronavirus disease 2019
CVIC tool: COVID-19 Vaccine Introduction and deployment Costing tool
EPI: Expanded Programme on Immunization
EUL: Emergency Use Listing Procedure
Lao PDR: The Lao People’s Democratic Republic
MoH: Ministry of Health
NDVP: National Deployment and Vaccination Plan
SAGE: Strategic Advisory Group of Experts on Immunization
USAID: United States Agency for International Development
WHO: World Health Organization
WPRO: Regional office for the Western Pacific
