# Supplementary material for: Estimating the delivery costs of COVID-19 vaccination using the COVID-19 Vaccine Introduction and deployment Costing (CVIC) tool: the Lao People’s Democratic Republic experience
Source: BMC Med. 2023 Jul 10;21:248. doi: 10.1186/s12916-023-02944-1 (PMC10332011; doi:10.1186/s12916-023-02944-1)
Supplement: Supplementary file 1 — Additional file 1: Appendix 1. Timeline for the development and key milestones of the COVID-19 Vaccine Introduction and deployment Costing (CVIC) tool. Appendix 2. Prepopulated data and sources in the COVID-19 Vaccine Introduction and deployment Costing (CVIC) tool. [file 12916_2023_2944_MOESM1_ESM.pdf]

Appendices of "Estimating the delivery costs of COVID-19 vaccination using the COVID-19 Vaccine Introduction and deployment Costing (CVIC) tool: the Lao People's Democratic Republic experience"

Authors: Karene Hoi Ting YEUNG, Eunkyoung KIM, YAP Wei Aun, Chansay PATHAMMAVONG, Lauren FRANZEL, Yu Lee PARK, Peter COWLEY, Ulla Kou GRIFFITHS, Raymond Christiaan W. HUTUBESSY

DOI: 10.1186/s12916-023-02944-1

Contents

Appendix 1. Timeline for the development and key milestones of the COVID-19 Vaccine Introduction and deployment Costing (CVIC) tool 2

Appendix 2. Prepopulated data and sources in the COVID-19 Vaccine Introduction and deployment Costing (CVIC) tool 3

Appendix 1. Timeline for the development and key milestones of the COVID-19 Vaccine Introduction and deployment Costing (CVIC) tool

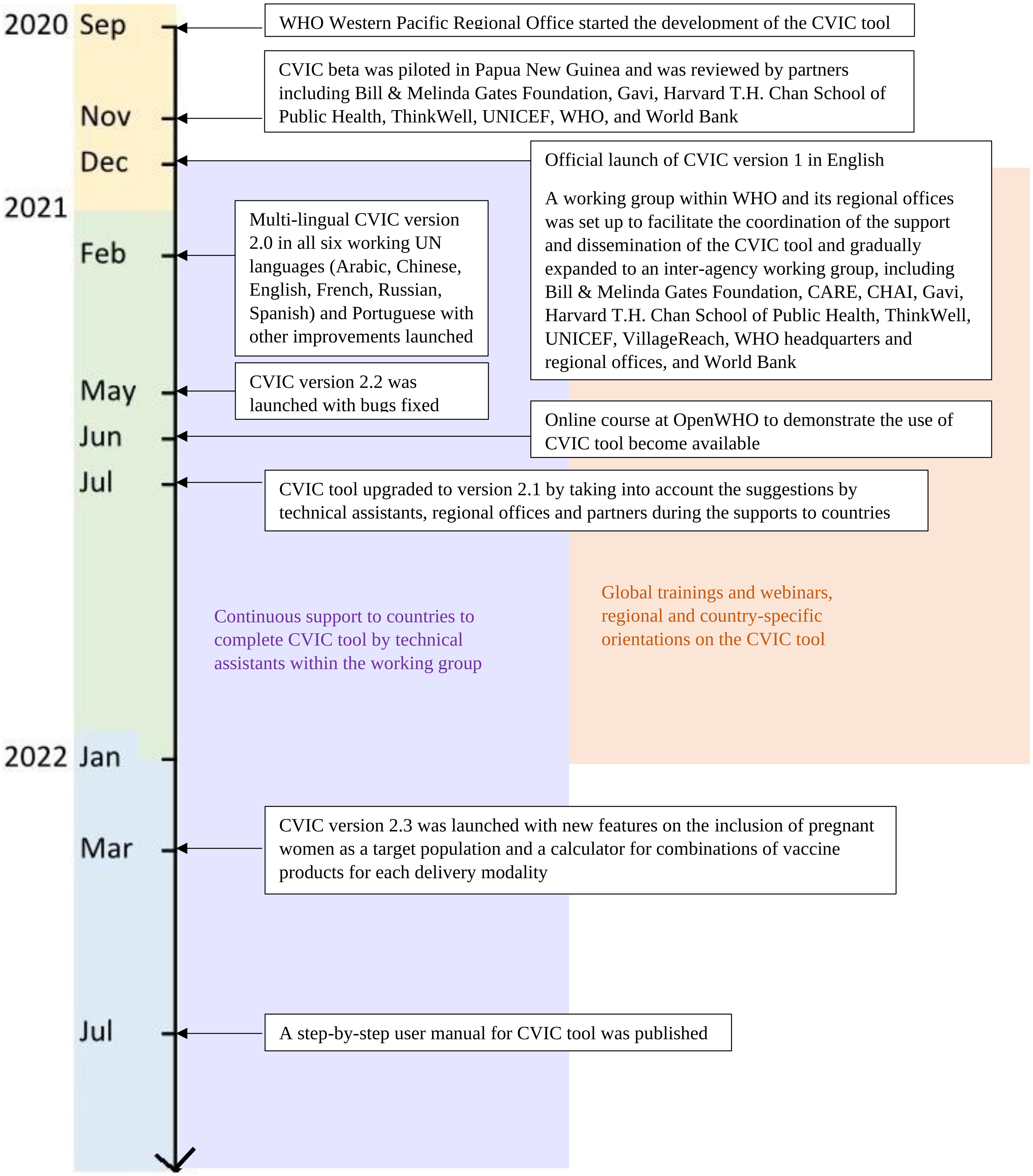

Appendix 2. Prepopulated data and sources in the COVID-19 Vaccine Introduction and deployment Costing (CVIC) tool

For user convenience and consistency, key parameters from global databases were compiled and included as overridable defaults in the CVIC tool.

Data	Source
Covid-19 vaccine specifications	WHO https://www.who.int/tools/covid-19-vaccine-introduction-toolkit#vaccine%20specific%20resources
Country-level health financing data	WHO Global Health Expenditure Database - Preliminary Data for 2018
Country-level population and demographic data	United Nations 2019 Revision of World Population Prospects; World Development Indicator (SP.POP.TOTL) for 2020
Crude birth rate	World Development Indicator (SP.DYN.CBRT.IN) for 2018
Gross monthly salaries for human resources for health	Serje, Juliana, Melanie Y. Bertram, Callum Brindley, and Jeremy A. Lauer. 2018. "Global Health Worker Salary Estimates: An Econometric Analysis of Global Earnings Data." Cost Effectiveness and Resource Allocation 16 (1): 10. https://doi.org/10.1186/s12962-018-0093-z . WHO-CHOICE. Updated to 2020.
Supply of human resources for health	WHO estimation of stock in 2020 based on latest reported health workforce stock in the National Health Workforce Accounts
Maximum redeployable human resources for health	Boniol M et al. Estimating the health workforce requirements and costing to reach 70% COVID-19 vaccination coverage by mid-2022: a modelling study and global estimates. BMJ Open 2022;12:e063059. doi: 10.1136/bmjopen-2022-063059)
Country context and other miscellaneous parameters<br><br>- Foreign currency exchange rates<br><br>- Country surface area<br><br>- WHO region<br>- World Bank income group, World Bank lending category, Pacific Island State status, Highly Indebted Poor Country status, and Fragile and Conflict Affected Situation status<br>- Membership of international bodies	 <br><br>- FloatRates (aggregated from central banks) http://www.floatrates.com/daily/usd.xml<br>- World Development Indicator (AG.SRF.TOTL.K2) for 2018<br>- WHO<br>- World Bank<br><br><br><br><br>- Relevant body or initiative
Data sources which are used for user informational purposes but are not used as defaults in estimations:<br><br>- Health infrastructure<br><br>- Diabetes prevalence (% of population ages 20 to 79)<br>- Prevalence of prevalence of obesity among adults, BMI >= 30 (crude estimate) (%)<br>- Urbanization<br><br>- Household size	 <br><br><br>- WHO Global Health Observatory (as of October 2020)<br>- World Development Indicator (SH.STA.DIAB.ZS) for 2019<br>- WHO Global Health Observatory (as of October 2020)<br><br>- World Development Indicator (SP.URB.TOTL.IN.ZS) for 2019<br>- United Nations, Department of Economic and Social Affairs, Population Division (2019). Database on Household Size and Composition 2019. https://population.un.org/Household/index.html
